# Single-marker and multi-marker mixed models for polygenic score analysis in family-based data

**DOI:** 10.1186/1753-6561-8-S1-S63

**Published:** 2014-06-17

**Authors:** Nora Bohossian, Mohamad Saad, Andrés Legarra, Maria Martinez

**Affiliations:** 1 Inserm UMR1043-CPTP, CHU Purpan, Toulouse, 31024, France; 2 University of Toulouse III-Paul Sabatier, Toulouse, 31062, France; 3 Inra UR631-SAGA, Castanet-Tolosan, 31326, France

## Abstract

Genome-wide association studies have proven successful but they remain underpowered for detecting variants of weaker effect. Alternative methods propose to test for association by using an aggregate score that combines the effects of the most associated variants. The set of variants that are to be aggregated may come from either of two modeling approaches: single-marker or multi-marker. The goal of this paper is to evaluate this alternative strategy by using sets of single-nucleotide polymorphisms identified by the two modeling approaches in the simulated pedigree data set provided for the Genetic Analysis Workshop 18. We focused on quantitative traits association analysis of diastolic blood pressure and of Q1, which served to control the statistical significance of our results. We carried out all analyses with knowledge of the underlying simulation model. We found that the probability to replicate association with the aggregate score depends on the single-nucleotide polymorphism set size and, for smaller sets (≤100), on the modeling approach. Nonetheless, assessing the statistical significance of these results in this data set was challenging, likely because of linkage because we are analyzing pedigree data, and also because the genotypes were the same across the replicates. Further methods need to be developed to facilitate the application of this alternative strategy in pedigree data.

## Background

Genome-wide association studies (GWAS) have proven successful in identifying common single-nucleotide polymorphisms (SNPs) associated with complex traits, but the underlying genetic architecture of these traits remains largely unknown. This classical approach is restricted to analyzing one SNP at a time and only those reaching genome-wide significance (α ≤1 × 10^−8^) are retained for further analyses. As such, for many complex traits, only a few SNPs have been identified, leaving a large part of the trait heritability unexplained. This is partially because a wide spectrum of effects may be implicated, many of which are not detected at the stringent significance criteria levels set in GWAS. One way to circumvent this limitation has been through using larger sample sizes, thus increasing the power to detect SNPs of weaker effect. Following their successful application in a few studies [1,2], attention has been turned to the use of alternative methods that propose to aggregate the effects of several SNPs into a polygenic score (PS) and test the PS for association with the trait. Typically, the PS is constructed in two steps. First, the set of SNPs to be included in the score is selected. The criteria for SNP selection vary between studies, but it is crucial that this set of SNPs contains only independent variants to avoid overrepresenting the same signal. Furthermore, because we are working in the context of detection rather than prediction, this set of SNPs must exclude all established variants, as they would drive the association of the PS, with the trait masking the weaker effects that we are looking to detect. Second, the reference alleles of these variants are combined in an unweighted or weighted manner. The unweighted approach assumes that all SNPs have the same effect size, which oversimplifies the context we are trying to evaluate (a mixture of different effect sizes). In the weighted approach, each reference allele is weighted by its effect estimated in an independent data set. The effect estimates could be obtained through a classical single-marker analysis whereby each effect is estimated one at a time or, alternatively, through a multi-marker analysis whereby all effects are estimated simultaneously. Studies suggest that the multi-marker analysis may outperform single-marker analysis in detection [3]. The goal of this study is to compare the PS analysis using sets of SNPs derived from single-marker and multi-marker analyses and to evaluate the value of this novel analytical approach, with the intent of shedding light on the true genetic architecture of a complex quantitative trait in family-based data.

## Methods

### Data and phenotypes

We used the pedigree data set provided for the Genetic Analysis Workshop 18 (GAW18) with knowledge of the simulated model. We focused on the simulated quantitative trait diastolic blood pressure measured at exam 1 (DBP_1). We used the trait Q1 to control for type I error. In the simulated model, there were 1457 SNPs (in 288 genes) contributing to DBP and/or systolic blood pressure variability. Their individual contribution ranged from as low as 0.001% for gene *ZZEF1 *to as high as 6.5% for gene *MAP4* (for DBP). Part of the total heritability was generated using polygenic alleles from 1000 SNPs that were randomly selected in each replicate. The trait Q1 was uninfluenced by any of the provided genotyped SNPs. There were 200 replicates but the genotypes were the same across the replicates.

We adjusted the traits for age and sex in a linear regression framework. Let $Y_{i}$ denote the trait adjusted for age at exam 1 and sex for individual *i *(i=1,…,N individuals). We used the full SNP map (j=1,…,J;J=8,348,674 SNPs). We denote the observed N×J genotype matrix by *X*. All genotypes were coded under the additive genetic model. We worked with models that treated the SNP effects as fixed or random. We used *β *to denote the J×1 vector of fixed SNP effects and *α *to denote the J×1 vector of random SNP effects. Finally, let *u *be the N×1 vector of random polygenic effects with $u\sim N\left(0,\sigma_{u}^{2}K\right)$ where *K *is twice the N×N kinship coefficient relationship matrix based on pedigree information and let *ϵ *be the N×1 vector of random residual effects with $\epsilon \sim N\left(0,\sigma_{\epsilon }^{2}I\right)$ where *I *is the N×N identity matrix.

### Single-marker mixed linear model (analysis limited to the SNPs and genes associated with DBP)

We first estimated the power to detect association of *Y *with any of these associated variants using the measured genotype test [4] (mixed-linear regression model), as implemented in QTDT software (http://www.sph.umich.edu/csg/abecasis/QTDT/).

The single-marker measured genotype test was conducted for each SNP, using all 200 replicates. We found that *MAP4 *was the only gene detectable (power = 96%) at the genome-wide significance level (*α*<1 × 10^−8^), which accounts for the largest percentage of the variance of DBP and contains SNPs with very strong individual effects. Any of the remaining SNPs or genes were unlikely to be detected at stringent significance criteria (power <50%). We estimated that it would take approximately 40 days to analyze the whole-genome data (>8 million SNPs) by replicate and by phenotype using the measured genotype test. Because of these computational constraints, we derived a new trait adjusted for family relatedness using GRAMMAR [5] as implemented in the GenABEL add-on package developed for the R statistical software [6], which allowed us to use the single-marker linear model. Lastly, because our goal was to evaluate whether power to detect association with SNPs with weak effects could be enhanced by pooling their effects, we further adjusted in a linear regression framework the decorrelated trait DBP_1 for the strong effects of *MAP4 * (SNPs3_48040283 and 3_48064367).

### Single-marker linear model

For the decorrelated trait we tested for association using simple linear model (without the random polygenic component) with PLINK version 1.07 [7].

### Polygenic score

Polygenic scores (PS) were built as follows:

(1) $$PS_{i}=\overset{S}{\underset{s=1}{\sum }}{\hat{\gamma }}_{s}X_{is}$$

where $PS_{i}$ is the polygenic score for the *i*^th^ individual, *S *is the size of the set of SNPs to combine, ${\hat{\gamma }}_{s}$ is the estimated effect of SNP *s *in a discovery data set, and $X_{is}$ is the number of minor alleles of the SNP *s *for the *i*^th^ individual in an independent data set (replication). The PS is computed after excluding genome-wide significant SNPs and including only independent SNPs (not in linkage disequilibrium). The PS values were computed with PLINK using the "-score" option. By default, if missing, the number of reference alleles was imputed from the sample allele frequency.

### Multi-marker mixed linear model

Here, all SNP effects are estimated simultaneously. For the original traits not adjusted for family relatedness (*Y*), the model is formulated as follows (using matrix notation):

(2) Y=μ1+Xβ+u+ϵ

where *μ *is the fixed mean effect and 1 is a vector of 1s. Because the above model analyzes all *J *markers jointly, it can account for the covariance structures between individuals through the realized genome-based relationship matrix [8] and can be formulated equivalently as a random regression approach as follows:

(3) Y=μ1+Xα+ϵ

where *α *is now a J×1 vector of *random* SNP effects with $\alpha \sim N\left(0,\sigma_{\alpha }^{2}I\right)$.

This is the widely used Best Linear Unbiased Predictor (BLUP) model (with only 1 fixed effect) [9]. We worked in a penalized regression framework ($l_{2}$ penalty) setting the penalty parameter, *λ*, as $\lambda =\sigma_{\epsilon }^{2}/\sigma_{\alpha }^{2}$. We derived $\sigma_{\alpha }^{2}$ from the relationship $V_{A}=\overset{J}{\underset{j=1}{\sum }}2p_{j}q_{j}\sigma_{\alpha }^{2}$, where $V_{A}$ is the total additive genetic variance, $p_{j}$ is the minor allele frequency, and $q_{j}=1-p_{j}$[10]. These analyses were carried out using the GS3 software (http://snp.toulouse.inra.fr/~alegarra/).

### Polygenic score analyses

Here, we were interested in comparing PS analysis with sets of SNPs derived from single-marker and BLUP models. We used replicate 1 as the discovery data set and each of the remaining 199 replicates to replicate the association of PS with the analyzed trait. Under the single-marker model, all SNPs were ranked by their *p* values. Under BLUP, all SNPs were ranked by the magnitude of their effect estimate. The best ranked *S *SNPs were used for computing the PS values. Here, we let *S *vary as 10, 50, 100, 1000, 5000, and 10,000 SNPs. To ensure that *S *contained only independent SNPs, the best SNP over a window of 100 kilobases (kb) was retained until the full SNP map was covered. (We also considered larger window sizes of 1 megabase (Mb) and 5Mb, but the results were similar and are not reported here.) These independent SNPs were then ranked. We conducted the same analyses on Q1. Furthermore, we also evaluated the association of PS, but this time by permuting DBP_1 within families in each replicate.

## Results and discussion

**Figure 1:**
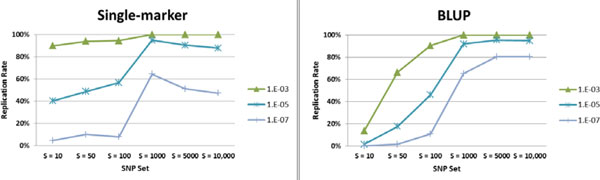
**Polygenic score association results on DBP_1**. Percentage of replicates (out of replicates 2 through 200) with significant evidence of association of PS with DBP_1 at a given nominal *p *value by SNP set *S *derived using either single-marker or BLUP strategies in replicate 1.

Figure 1 illustrates the PS association results using the two strategies, single-marker and BLUP, for selecting the top *S *SNPs in replicate 1. The results are expressed as the percentage of replicates (out of replicates 2 through 200, hereinafter referred to as the replication rate) with significant evidence of association of PS with DBP_1 at different nominal *p* values and by SNP set size *S*. With the single-marker strategy, the replication rates tend to increase with the SNP set size until reaching a peak at S=1000 SNPs, after which they begin to decline, especially at stringent significance criteria levels (*p*value ≤1E-05). (This may be happening as a result of the increased noise with the larger *S* [>1000].) For the BLUP strategy, the peak is reached at S=5000 SNPs, after which the replication rates tend to remain stable. Irrespective of the strategy, however, the replication rates are rather high, especially at nominal *p* values ≥1 × 10^−3 ^and for larger set sizes (S ≥1000) where they are nearly always at 100%. For smaller set sizes (S ≤100), replication rates are greater under the single-marker strategy than under the BLUP strategy. The opposite trend is observed for larger set sizes (S ≥5000).

To evaluate the significance of these findings, we performed the PS association analyses on Q1. Note that because of the small number of available replicates (199), replication rates could not be estimated at stringent criteria levels (ie, nominal *p *values <1%). Estimates of the replication rates were close to the theoretical values, whether the top SNPs were selected under the single-marker approach or the BLUP approach, and irrespective of set size *S *(ranging from 5% to 8% at *p* value = 5%). PS association analyses conducted on the permuted DBP_1 trait yielded to slightly inflated rates (results not shown), especially for larger set sizes *S* and under the BLUP strategy (ranging from 8% to 12% and from 6% to 17% under single-marker and BLUP strategies, respectively). From these results it is not clear whether the distribution of the PS association test appropriately follows the theoretical distribution in the pedigree data set even though the traits were decorrelated.

## Conclusions

Using the measured genotype test, a classical approach to detect association in family data, we found that, with the exception of the SNPs in the *MAP4 *gene, it had no power to detect SNPs of weaker effect at the genome-wide significance level. In our study, we aimed to evaluate PS association analysis as a method to detect SNPs of weaker effect that fail to reach genome-wide significance in classical GWAS. We used a single-marker approach and a multi-marker approach to derive the top SNP sets. In summary, both strategies lead to relatively high replication rates, especially when large sets of SNPs (≥1000) were considered. Our study presents some weaknesses and limitations. PS analysis was conducted using linear regression on the decorrelated traits. Thus, either the way we constructed the sets of independent top SNPs or the fact that the genotypes were the same in all replicates may have led to biased and inflated estimates of power rates. Type I error rates were found close to the theoretical values when analyzing Q1, but not when analyzing the permuted DBP trait. It appears, therefore, that linkage may have affected our PS analyses even if we worked with decorrelated traits. These results suggest that the presently available methods need to be extended to address the challenges of PS association analyses in pedigree data.

## Competing interests

The authors declare that they have no competing interests.

## Authors' contributions

MM designed the overall study; AL contributed to the design and implemented the software for the BLUP model; NB, MS, and MM conducted statistical analyses and drafted the manuscript. All authors read and approved the final manuscript.
